# Lattice Defects Engineering in W-, Zr-doped BiVO_4_ by Flame Spray Pyrolysis: Enhancing Photocatalytic O_2_ Evolution

**DOI:** 10.3390/nano11020501

**Published:** 2021-02-16

**Authors:** Panagiota Stathi, Maria Solakidou, Yiannis Deligiannakis

**Affiliations:** Laboratory of Physics Chemistry of Materials & Environment, Department of Physics, University of Ioannina, 45110 Ioannina, Greece; pstathi@cc.uoi.gr (P.S.); maria-sol@windowslive.com (M.S.)

**Keywords:** BiVO_4_, W-doping, Zr-doping, flame spray pyrolysis, oxygen vacancies, defects, Raman, XPS, photocatalysis, water splitting, O_2_

## Abstract

A flame spray pyrolysis (FSP) method has been developed, for controlled doping of BiVO_4_ nanoparticles with W and Zr *in tandem* with the oxygen vacancies (Vo) of the BiVO_4_ lattice. Based on XPS and Raman data, we show that the nanolattice of W-BiVO_4_ and Zr-BiO_4_ can be controlled to achieve optimal O_2_ evolution from H_2_O photocatalysis. A synergistic effect is found between the W- and Zr-doping level in correlation with the Vo-concentration. FSP- made W-BiVO_4_ show optimal photocatalytic O_2_-production from H_2_O, up to 1020 μmol/(g × h) for 5%W-BiVO_4_, while the best performing Zr-doped achieved 970 μmol/(g × h) for 5%Zr-BiVO_4_. Higher W-or Zr-doping resulted in deterioration in photocatalytic O_2_-production from H_2_O. Thus, engineering of FSP-made BiVO_4_ nanoparticles by precise control of the lattice and doping-level, allows significant enhancement of the photocatalytic O_2_-evolution efficiency. Technology-wise, the present work demonstrates that flame spray pyrolysis as an inherently scalable technology, allows precise control of the BiVO_4_ nanolattice, to achieve significant improvement of its photocatalytic efficiency.

## 1. Introduction

Since the 1972 report by Fukushima and Honda [1] on the photocatalytic water splitting using TiO_2_, several other types of semiconductors have been evaluated as photocatalysts. Tungtates [2], vanadates molybdates and niobates [3] have been found to be efficient photocatalysts for O_2_ evolution from H_2_O. So far, among the most efficient O_2_-evolving photocatalysts IrO_2_ stands-out as the best [3] however its high-cost is prohibitive. Currently O_2_-produciton efficiencies for IrO_2_ photocatalysts are reported to be in the range 5000–7000 μmol/(g × h) [3]. TiO_2_ as a reference material has been extensively studied for O_2_-evolution photocatalysis [1,3]. So far O_2_-produciton efficiencies for TiO_2_ photocatalysts are in the range 50–200 μmol/(g × h) [4]. BiVO_4_ is among the most promising O_2_-evolving photocatalysts, i.e., due to suitable narrow band-gap energy (Eg), i.e., 2.2–2.4 eV and high optical adsorption efficiency. Monoclinic scheelite BiVO_4_ is an *n*-type semiconductor with a direct band gap of 2.4 eV, thus it absorbs visible light, λ = 420–530 nm with an optical penetration depth of l_p_ = 100–500 nm [4]. BiVO_4_ can be used in Z-scheme photocatalysts [4], i.e., to heal low mobility of photogenerated charge carriers in BiVO_4_ and high recombination rates of photogenerated electron-hole pairs [5].

So far, there is credible evidence that doping of BiVO_4_ can be an efficient strategy to improve the photocatalytic performance [6,7,8,9,10,11,12,13,14,15,16,17,18,19]. Highly encouraging results show that appropriate doping of BiVO_4_, i.e., with Mo [7], W [8,9], P [10], B [11] or Ce [12] can result in significant enhancement of O_2_-evolution. Currently, all reported methods for synthesis of doped BiVO_4_ photocatalysts, concern liquid-chemistry methods. For example Ikeda et al. [13] have synthesized Zr-doped BiVO_4_ photocatalysts, using a coprecipitation method, and their material exhibited O_2_ evolution 500 μmol/(g × h) [15]. A W-doped BiVO_4_ catalyst had achieved O_2_ production of 665 μmol/(g × h)[14]. In 2012, Lee and coworkers [15] had compared the O_2_-evolution efficiency for 12 types of different metal-ion dopants in BiVO_4_, prepared by the same solid-state reaction process. Among the tested dopants, only W and Mo showed a dramatic enhancement of O_2_ evolution activity, i.e., 600 μmol/(g × h) and 2000 μmol/(g × h) respectively vs. 100 μmol/(g × h)for undoped BiVO_4_. Cerium-doped BiVO_4_ prepared with a solvothermal method [19] achieved O_2_ production of 325 μmol/(g × h).

Flame spray pyrolysis (FSP) is an one-step flame-process synthesis [16], which allows engineering of nanoparticles with well-controlled composition, phase-purity, crystallinity and size [17]. Originally, FSP synthesis of BiVO_4_ has been reported by Amal, Madder and Kudo [18]. In [18] it was found that when the FSP-made BiVO_4_ contains excess of lattice-defects, this is detrimental for photocatalytic O_2_ production. Thus, the authors had developed a post-FSP liquid-phase treatment [18] to optimize the photocatalytic performance of BiVO_4_, i.e., achieving 300 μmol/(g × h) O_2_ per gram of BiVO_4_ per hour. Their post-FSP protocol included aqueous acid-treatment with addition of bismuth (Bi) and vanadium (V) atoms, which was shown to promote the formation of photo catalytically active scheelite-monoclinic BiVO_4_ phase. On the other hand, theoretical calculations [19] show that oxygen-vacancies, if appropriately engineered, can be beneficial for photocatalytic O_2_ production. More specifically, a distinct role of surface-oxygen vacancies vs. bulk-oxygen vacancies have been dictated by DFT calculations for pure BiVO_4_ [19]. According to Wang et al. [19], photoinduced polarons formed from O-vacancies in the bulk can contribute to conductivity, while those at the surface might have an opposite effect. Intriguingly, it has been suggested that surface O-vacancies might have a beneficial effect on O_2_-adsorption on BiVO_4_ during photocatalysis, i.e., according to the theoretical study in [19]. Another theoretical study indicated that the fine-balance between surface and bulk O-vacancies should be considered carefully in the quest of optimal O_2_ photoproduction by BiVO_4_ [20].

The pivotal role of O-vacancies has also been reported for doped-BiVO_4_ also. Specifically, in W-doped BiVO_4_ [20]_,_ theoretical calculations indicate that a localized-state formed inside the band gap in W-doped BiVO_4_ containing oxygen vacancies [20] can serve as a recombination center, thus it lowers the photoinduced charge-separation efficiency [20]. In [20] it was predicted that better performance can be achieved by introducing oxygen vacancy *on the surface* of a W-doped BiVO_4_, simultaneously avoiding oxygen vacancy *in the bulk* [20]. The same trend seems to be true for Mo-doped BiVO_4_ [7]. DFT calculations indicate that surface oxygen quasi-vacancies enhance O_2_ photoproduction in Mo-doped monoclinic BiVO_4_ by facilitating separation of photoinduced carriers [7].

Herein, we used flame spray pyrolysis as a method to engineer W- and Zr-doped BiVO_4_ with controlled O-vacancies’ content. Using XPS and Raman spectroscopies we have studied the interrelation between W- or Zr-doping and O-vacancies, in conjunction with photocatalytic O_2_ evolution. Thus, the main aims of the present study are: (i) the development FSP protocols for synthesis of doped-BiVO_4_ with controlled W- and Zr-dopant content, (ii) to optimize the nanomaterials’ photocatalytic O_2_ production from H_2_O oxidation, and (iii) to discuss the underlying catalytic mechanism, revealing the crucial role of oxygen vacancies in conjunction with W and Zr.

## 2. Materials and Methods

All solvents used were of commercial grade and have been purchased from Sigma Aldrich. Bi and V metal-organic precursors, i.e., bismuth (III) nitrate pentahydrate (Bi(NO_3_)_3_) (99% purity) and vanadium(V) oxytriisopropoxide (OV(OCH(CH_3_)2)_3_) (99% purity) respectively. For the dopings, the W- and Zr-precursors used were ammonium metatungstate hydrate (85% purity) and zirconium (IV) isopropoxide isopropanol complex (99.9% purity) obtained from Strem Chemicals.

### 2.1. Flame Synthesis of Nanocatalysts

The lab-scale FSP reactor used for the synthesis of nanocatalysts has been described recently [21]. The bismuth precursor was prepared by dissolving Bi-nitrate in (triethylene glycol dimethyl ether/acetic acid (70/30 *v*/*v*)) (0.5 M) and sonicated for 30 min at 50 °C. The V-precursor was prepared by dissolving vanadium-oxytriisopropoxide in xylene (0.5 M). For the doping materials, ammonium metatungstate hydrate or zirconium (IV) isopropoxide was added in the Bi/V mixture solution at the nominal atom ratios, as specified in Table 1. To produce the particles in FSP, a 1:1 mixture of Bi:V solution was fed, atomized through a 300 μm capillary at 5 mL/L and dispersed by 5 L/min O_2_ (Linde, purity > 99%). A self-sustained O_2_:CH_4_ (5 L/min and 1.5 L/min) pilot-flame was used to initiate combustion. Pressure-drop at the nozzle tip was fixed at 2 bar, and an additional 5 L/min sheath-O_2_ was used. The product powder was collected, using a vacuum pump (Busch V40), on a glass microfiber filter (Albet). All prepared materials studied herein, are listed in Table 1. For the sake of simplicity, in Table 1 and throughout the text, the samples were code-named according to their nominal % dopant loading, i.e., %W or % Zr in the precursor-solution, and their synthesis configuration. As shown previously [18], adjusting the particle-collection filter to higher temperatures > 340 °C promotes the in-situ formation of monoclinic scheelite BiVO_4_ phase [18]. Accordingly, in our set-up the FSP particle-collecting filter temperature was adjusted to be 350–360 °C.

### 2.2. Characterization of Nanocatalysts

X-Ray Diffraction (XRD): The crystal structures of the nanocatalysts were analyzed by XRD in a Bruker Advance D8 diffractometer (Cu Ka radiation λ = 1. 5406 Å, 40 kV, 40 mA) at 2θ = 10–60° (step size of 0.03° at a rate 2 s per step). The average crystallite sizes of BiVO_4_-based particles were calculated by the Scherrer Equation (1):

(1) $$d_{XRD=}\frac{k\lambda }{\beta \left(\cos\theta \right)}$$

where *d_XRD_* is the crystallite size (nm), k is a shape constant (in this case k = 0.9), *λ* is the wavelength of Cu Kα radiation (1.5406 Å), *β* is the full-width at half- maximum and *θ* is the peak-diffraction angle.

Brunauer–Emmett–Teller (BET) Analysis: The specific-surface-area (SSA, m^2^/gr) of the synthesized materials was determined by the N_2_ adsorption–desorption method at 77 K using a Quantachrome Autosorb-1 instrument (Bounton Beach, FL, USA). In order to acquire the BET isotherms, powders were degassed for 4 h at 120 °C in flowing N_2_ over a relative pressure range of P/P_0_ = 0–1.

X-ray Fluorescence (XRF): Sample excitation was performed with an annular 109Cd radio-isotopic source (RITVERC GmbH). The source has a radius of 12.5 mm and is housed in a cylindrical container, fixed coaxially above a CANBERRA SL80175 Si(Li) detector (5 mm crystal thickness, 80 mm^2^ area), with a 25 μm-thick Be window and an energy resolution f 171 eV for the 5.9 keV Mn Kα line. Data acquisition was performed using a PCI card, controlled by the ORTEC MAESTRO-32 software, and spectral analysis was carried out using the WinQxas software package (International Atomic Energy Agency Laboratories Seibersdorf, XRF Group, Seibersdorf (Austria), IAEA 1997–2002).

Raman Spectroscopy: Raman spectroscopy measurements were performed in a HORIBA XploRA PLUS instrument, which employed a 785 nm diode laser as excitation source focused with a microscope. The samples were pressed into pellets and placed on a glass plate. The spectra were recorded for 10 s with 30 accumulations in order to obtain adequate signal-to-noise ratio.

TEM: The morphology of the samples was analyzed by high-resolution transmission electron microscopy (HRTEM) using a Philips CM 20 microscope operated at 200 kV with 0.25 nm resolution. Before the measurements, the samples were ground in a mortar and dry loaded onto a support film (Lacey Carbon, 300 mesh, (Cu)). Recorded images were analyzed by Gatan Digital Micrograph software. Particle-size was calculated using the ImageJ software.

X-ray photoelectron spectroscopy (XPS) data were acquired in a surface analysis ultrahigh vacuum system (SPECS GmbH) equipped with a twin Al-Mg anode X-ray source and a multichannel hemispherical sector electron analyzer (HSA-Phoibos 100). The base pressure was 2–5× 10^−9^ mbar. A monochromatized Mg-Kα line at 1253.6 eV and analyzer pass energy of 20 eV were used in all XPS measurements. The binding energies were calculated with reference to the energy of C1s peak of adventitious carbon at 284.5 eV. The peak deconvolution was performed using a Shirley background.

### 2.3. Catalytic Evaluation

The photocatalytic O_2_-evolution experiments were performed using an immersion-well reactor (Photochemical Reactors Ltd., Berkshire UK, Model 3210), provided with two angle sockets and one vertical socket of total reaction volume of 300 mL, being under tap water circulation, at constant temperature 25 ± 3 °C. Light source was an inlet medium-pressure mercury lamp (Model 3010,125 W, light output 7 × 10^18^ photons/sec). In each experiment, 50 mg of the catalyst was suspended into 150 mL milli-Q water, which contained 0.1 M NaOH (pH≅13.3) and 0.02 M Na_2_S_2_O_8_ (final concentration of catalyst 0.2 g/L). Before the reaction begins, the suspension was bubbled with Ar gas (99.997%) at least 1 h, in order to remove atmospheric gas. The experiments with Au were carried out using hydrogen-tetrachloroaurate(III)-trihydrate (HAuCl_4_·3H_2_O, 99.9%, Alfa Aesar, Kandel Germany ) as a precursor. Qualitative and quantitative monitoring of produced H_2_ was succeeded via a continuous online gas chromatography system combined with a thermo-conductive detector (GC-TCD Shimadzu GC-2014, Carboxen 1000 column, Ar carrier gas).

## 3. Results

### 3.1. Characterization of the FSP-Made BiVO_4_-Based Photocatalysts

**Figure 1:**
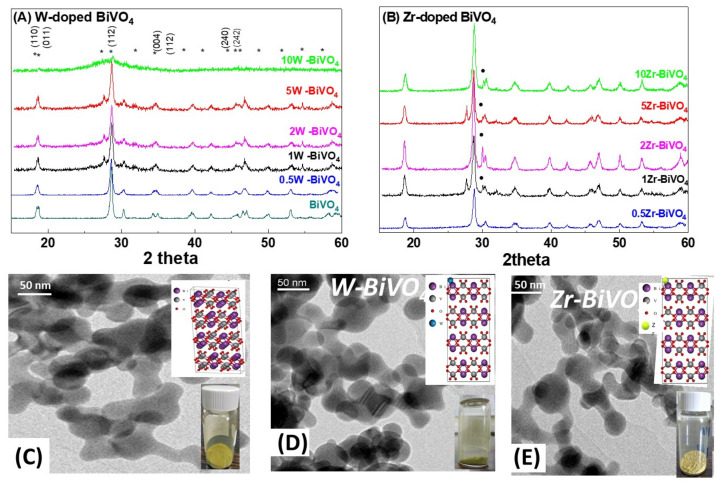
XRD patterns (upper row, W-doped (**A**) and Zr-doped (**B**)), and TEM images (lower row) for BiVO_4_ (**C**), 5W-BiVO_4_ (**D**) and 5 Zr-BiVO_4_ (**E**). Inserts in C-E: photos of the nanopowders and atomic structure of the materials. In figure also presented the miller index of BiVO_4_ structure. In (A), stars (*) mark the main Miller-planes of BiVO_4_. In (B) the dots (●) mark ZrO_2_ phase.

XRD: The X-Ray diffraction patterns for all FSP-prepared BiVO_4_ nanoparticles are presented in Figure 1. Pristine BiVO_4_ is also included for comparison. The coexistence of monoclinic-scheelite (see * marks) and tetragonal-scheelite phases is apparent in all cases (JCPDS card 75-2480, JCPDS 14-0133) respectively. The structural characterization results of the present nanocatalysts are summarized in Table 1.

It is known that the monoclinic-scheelite BiVO_4_ phase can be obtained from the irreversible phase transformation of tetragonal-zircon structure at temperatures > 400 °C [4,18]. In accordance with [18], in FSP-made pristine BiVO_4_, crystallization of monoclinic-scheelite occurred at temperatures >340 °C. In the XRD data in Figure 1, the peak-splitting of diffraction peaks at 2θ = 18.5, 35 and 46° evidence the presence of the monoclinic-scheelite phase, at all BiVO_4_ based nanomaterials. The XRD-derived particle sizes are listed in Table 1. In general, W- or Zr-doping caused rather minor changes in the particle sizes and SSA, see Table 1. In doped-materials, diffraction peaks from secondary phases such as BiWO_4_ at 2θ = 17.2° were detected [9]. Apart from this, W-doping had no effect on the BiVO_4_ peak positions, neither BiVO_4_ phase-change, i.e., from monoclinic to tetragonal. The present XRD data indicate that in W-doping up to 5% did not change the crystal structure of BiVO_4_. In contrary, a higher W-doping (10%) caused a major deterioration of the crystallinity, i.e., a broad XRD pattern is observed, see Figure 1A. Herein, this material was not further considered for photocatalysis.

In Zr-doped BiVO_4_, certain XRD peaks appeared to be affected, e.g., see the BiVO_4_ diffraction at 2θ = 18° in Figure 1B, while at Zr-doping > 1% diffraction peaks attributed to ZrO_2_ particles (back dots in Figure 1B) can be observed. Analysis of the XRD patterns (see Figure S1 in Supporting Information) shows that these corresponded to cubic-ZrO_2_ nanoparticles of 3 nm diameter.

The TEM images, Figure 1, show the formation of neck-sintered structures, which is in accordance with the original work for FSP-made BiVO_4_ [18]. Such neck-sintered aggregates formations has also been reported recently also for other Bi-based, i.e., BiFeO_3_ nanoparticles made by FSP [22].

**Figure 2:**
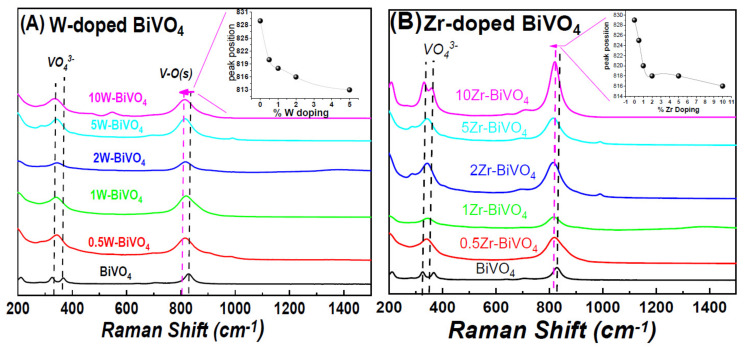
Raman spectra for the FSP-made BiVO_4_-based photocatalysts: (**A**) W-doped nanomaterials and (**B**) Zr-doped nanomaterials. Insert: shift of Raman V-O(s) peak at 830 cm^−1^.

Raman spectroscopy: Figure 2 illustrates Raman spectra for the FSP-made nanomaterials. In all the spectra, the characteristic vibration peaks from BiVO_4_ were detected of all materials_._ The peaks at 330 and 373 cm^−1^ were assigned to the asymmetric and bending vibrations of VO_4_^−3^ unit respectively [18]. The peak at 830cm^−1^, marked as V–O(s) in Figure 2, can be attributed to stretching vibrations of the V-O bonds in the VO_4_^−3^ unit [12]. The position of Raman peak at 830 cm^−1^ gives information on the V-O on length [12] thus is a good indicator for distortions of the overall lattice. In the doped BiVO_4_ materials, Figure 2, we observe that the V-O(s) band position at 830cm^−1^ is gradually downshifted, see inset in Figure 2A,B for increased W- and Zr-doping percentage respectively. These shifts indicate gradual deformation of VO_4_^−3^ unit, due to insertion of the W- or Zr-atoms into the BiVO_4_ crystal. Notice that these lattice deformations could only be probed by Raman, while they were not resolved in the XRD.

**Figure 3:**
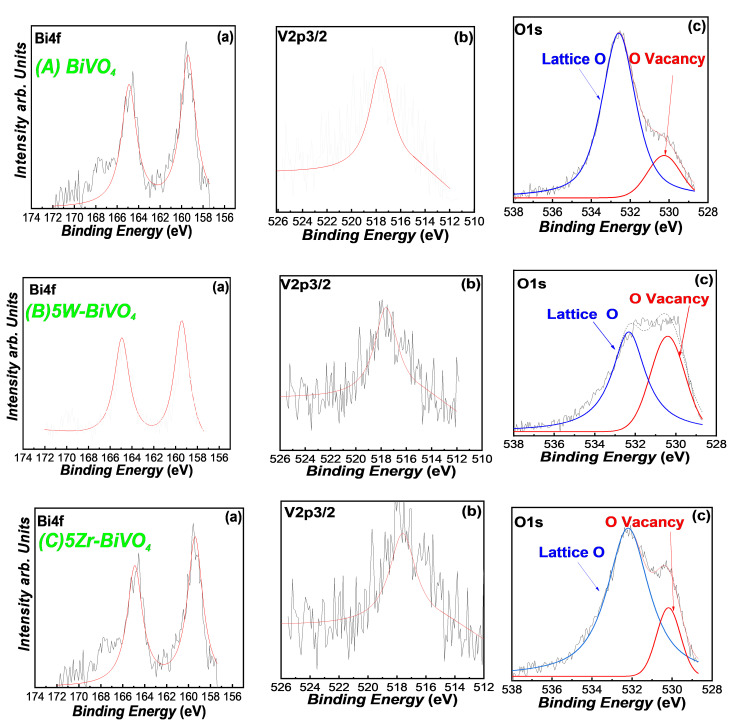
XPS spectra of (**A**) pristine FSP-made BiVO_4_, (**B**) 5 W-BiVO_4_ and (**C**) 5 Zr-BiVO_4_ ((**a**) Bi_4f_, (**b**) V_2p_ and (**c**) O_1S_).

X-ray Photoelectron Spectroscopy (XPS): Representative broad-scan XPS data for FSP-made BiVO_4_-based materials are presented in Figure 3. In all cases, the XPS data confirm the presence of Bi, V and O, while no peaks assignable to W or Zr can be discerned in the 5%W or 5%Zr materials. However, affirmative evidence for the presence of the dopant species is confirmed by our XRF spectroscopy data (see Table 1). The Bi_4f5/2_ and Bi_4f7/2_ binding energies at 164.2 and 158.7 eV respectively, were well resolved for all the photocatalysts. The V_2p3/2_ peak is also resolved in all cases. The O_1s_ binding energies can be fitted with two peaks, ascribed to the lattice oxygen of BiVO_4_ crystal, and oxygen vacancies formed on the surface of the BiVO_4_ [23,24].

Doping with W or Zr, resulted in an enhancement of the concentration of oxygen vacancies (Vo), see Figure 3. Thus, the XPS data in Figure 3, show that the as-prepared FSP-made BiVO_4_ contains O-vacancies, whose concentration can be promoted by W- or Zr-doping. This phenomenon, i.e., the correlation of W with O-vacancies sites, has been recently discussed in theoretical DFT studies [19,20,24]. More specifically, theoretical calculations indicate that surface O-vacancies enhance the electron density near the bottom of the conduction band of BiVO_4_ [19] Additionally, there is theoretical evidence [20,24] that O-vacancies may play a role in the interfacial charge transfer. Importantly, the DFT data entail that the role of W-doping is interlinked with the O-vacancies in the enhancement of the photocatalytic electron-hole separation [24]. As we show hereafter, our data on photocatalytic O_2_-evolution, provide corroborating experimental evidence for the positive effect of W- and Zr-doping in conjunction with the Vo in BiVO_4_.

**Figure 4:**
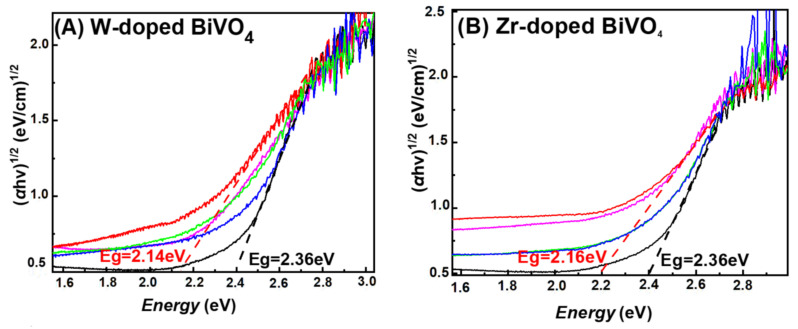
Kubelka–Munk transformed diffuse reflectance UV–Vis spectra of FSP-made BiVO_4_-based nanocatalysts. (**A**) W-doped BiVO_4_ and (**B**) Zr-doped BiVO_4._

Diffuse Reflectance UV–Vis (DR-UV-Vis): Figure 4 presents DR-UV–Vis spectra and optical band gap energy determination using the Kubelka–Munk [25] formula (2) where *α* is the absorption coefficient, and the n-value is related to the type of photoexcited transition, i.e., direct or indirect [25].

αhv = A(hv − E)^n/2^ (2)

In our analysis, a value of *n* = 2 was set, i.e., since BiVO_4_ is a direct band gap semiconductor [25].

All our FSP-made materials showed adsorption edges with a tail extending towards low-energies, which was enhanced at increasing W- or Zr-doping, see Figure 4A and Figure 4B respectively. Optical band gap energies estimated from the DR-UV–Vis data, are marked by the tangent lines in Figure 4, while the full list of E_g_ values are presented in Table 1. A band-gap value of E_g_ = 2.36 eV is typical for pristine BiVO_4_ [24]. Notice that pristine BiVO_4_ showed also a low-energy tail, i.e., resolved as a weak hump at 1.6 eV in Figure 4A. This can be attributed to low-energy photons absorbed via intraband states created by the oxygen-vacancies [24] at the Fermi level of BiVO_4_.

**Figure 5:**
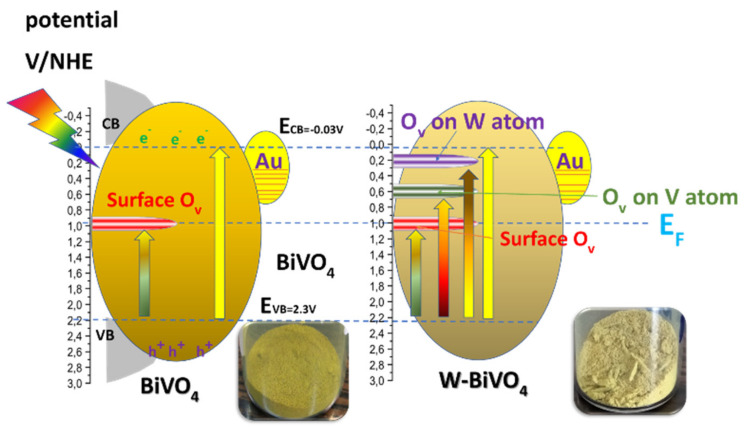
Schematic depiction of the energy levels of FSP-made photocatalysts. Surface O-vacancies create Fermi-level states in BiVO_4_ (**left**). In addition, W-doping (**right**) generates additional intraband states whose position depends on the relative location of W, V atoms and O-vacancies [24].

Figure 5 presents a schematic depiction of the energy profile of our BiVO_4_-based particles. Doping causes a progressive decrease of the E_g_ values, see Figure 4 and Table 1, i.e., the E_g_ was progressively decreased to 2.14 eV for 5W-BiVO_4_ and E_g_ = 2.16 eV for 5Zr-BiVO_4_. As analyzed previously in detail, for W-doped BiVO_4_ [19,20] these trends can be attributed to creation of intraband states, see Figure 5 (right). DFT calculations [5,19,20,24] indicate that the energy of the intraband states is sensitive to the exact location of the W-atom, i.e., whether an oxygen-vacancy occurs next to a W- or next to a V-atom [24], see Figure 5.

In our materials, in all cases, the E_g_ decrease was accompanied by enhanced light-absorbance at low-energies, verifying that such intraband states are indeed formed upon insertion of the W- or Zr-heteroatoms in to the BiVO_4_ lattice [24]. Notice that such low-energy light-absorbance is manifested in a more-opaque, less-sharp yellowish color of the W-BiVO_4_ and Zr-BiVO_4_ materials, see photos of the FSP-powders in Figure 5. Accordingly, we consider that in our FSP-made BiVO_4_, all the possible intraband states depicted in Figure 5 (right) might be formed, thus a quasi-continuum of low-energy photons are absorbed by the doped BiVO_4_ materials.

Overall, the present XPS, Raman and DRS-UV–Vis data provide converging evidence that: (i) our FSP-made BiVO_4_ contains oxygen vacancies. (ii) W-doping and Zr-doping increase the population of the oxygen vacancies. (iii) W-doping and Zr-doping generate intraband energy states, which enhance the photon-absorbance profile at increased wavelengths, i.e., lower energies down to 50% of the original E_g_. These observations are of immediate relevance to the photocatalytic O_2_-evolution, as we show in the following.

### 3.2. Catalytic Results

#### 3.2.1. Photocatalytic O_2_-Evolution from H_2_O

**Figure 6:**
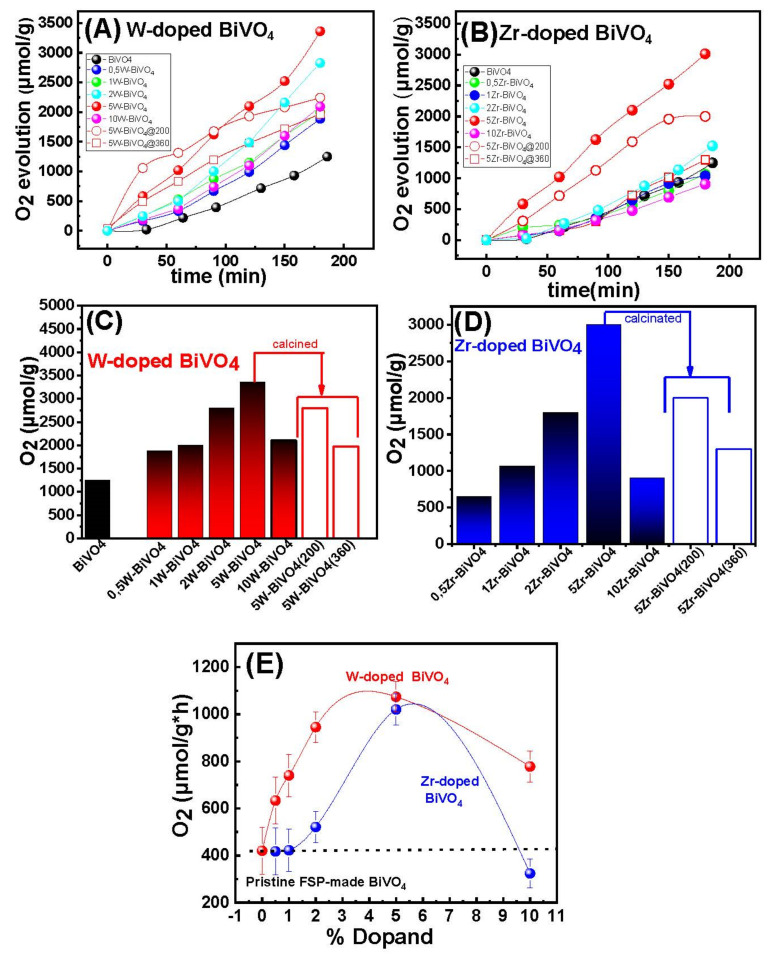
(**A**) Kinetics of O_2_-evolution by the W-BiVO_4_ and (**B**) O_2_ evolution by Zr-BiVO_4_. Solid symbols are for as prepared materials. Open symbols for calcined at 200 °C (open cycles), or 360 °C (open squares). (**C**) Comparison of O_2_-evolution efficiencies of the present FSP-photocatalysts after 180 min of photocatalytic reaction (W-BiVO_4_), (**D**) (Zr-BiVO_4_) and (**E**) O_2_ evolution rate vs. the dopant loading. The lines in (**E**) are for guiding the eye.

The photocatalytic water oxidation activity of our BiVO_4-_based catalysts was investigated, using Au as cocatalyst. Figure 6A,B presents data on the O_2_ evolution kinetics, for the W- and Zr-doped BiVO_4-_based catalysts.

The data in Figure 6 show that all photocatalysts produced O_2_ from H_2_O splitting. More precisely, after 180 min of irradiation, the O_2_ generated by the best-performing catalyst, 5W-BiVO_4_, was 2217 μmol per g of material, Figure 6A. The homologous material 5Zr-BiVO_4_ produced 3018 μmol/g within 180 min of reaction, Figure 6B. For comparison, Figure 6C,D presents the O_2_ evolution activities in (μmoles per gram of catalyst) at t = 180 min, based on the data of Figure 6A,B. The data are summarized in Table 2.

According to Figure 6E and Table 2, W-doping even at 0.5% resulted in a drastic improvement of the O_2_ evolution activity vs. pristine BiVO_4_. Among them, 5 W-BiVO_4_ exhibited a 270% higher activity than pristine BiVO_4_ under the same catalytic conditions. When we compared the W-doped vs. Zr-doped materials in Figure 6E, we noticed that significantly higher Zr-doping is required to achieve comparable O_2_ production rate, as for W-doping. Take as example the 0.5%-doped materials: O_2_ production rate was 633 μmol/(g × h) for 0.5% W doped vs. 235 μmol/(g × h) for the 0.5% Zr-doped BiVO_4_. Notice that the two materials had analogous specific-surface-area, i.e., 45 m^2^/gr vs. 47 m^2^/g, see Table 1. Thus, if we would normalize per SSA, the W-doped material far more active than the Zr doped BiVO_4_. In all cases, high dopant-loading, i.e., 10%W or 10%Zr, resulted to lower O_2_-evolution efficiency.

#### 3.2.2. Comparison with Literature

In Table 3 we listed the O_2_ evolution rates (μmol/g × h) reported so far for pertinent BiVO_4_ based photocatalysts, synthesized with various methods or doped with different ions.

The data in Table 3 show that the present 5W-BiVO_4_ and 5Zr-BiVO_4_ ranked among the best-performing O_2_-evolving photocatalysts. Taking into account the differences in the photon-flux, i.e., low-light 125 W of our irradiation system vs. 400 W in reference [26], further confirms that the present FSP-made materials stand at high performance rank. Our data exemplified that a concomitant control of O-vacancies and W-doping is a potent route for engineering of efficient low-cost O_2_-evolving photocatalysts.

#### 3.2.3. Reusability of the Nanocatalysts

Among the main aims of the heterogeneous catalysis is the reusability of the catalyst. In the present work, the reusability of our best-performing photocatalyst, 5W-BiVO_4_ was evaluated. After each catalytic use, the solid catalyst was recovered by centrifugation (6000 rpm, 15 min). The liquid supernatant was discarded, and the solid was used for a new reaction under the same catalytic conditions—with no further addition of catalyst.

**Figure 7:**
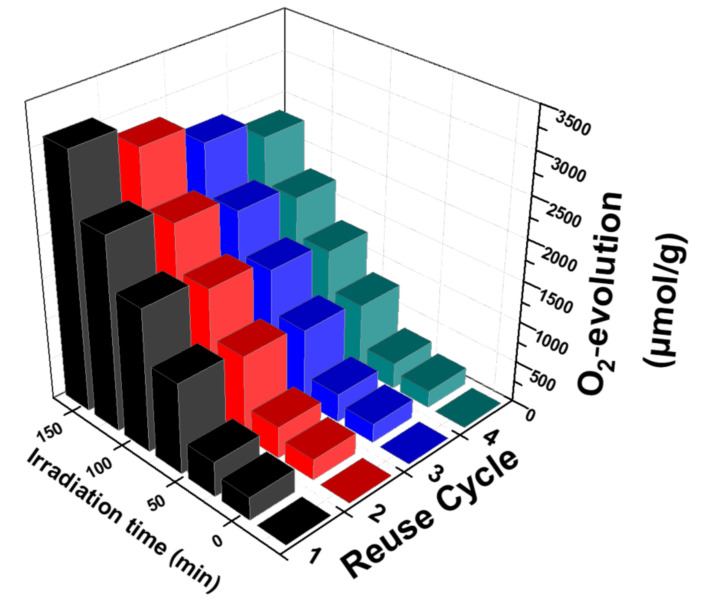
Photocatalytic O_2_ evolution by sequential reuse of the same 5W-BiVO_4_-batch.

According to Figure 7, after four sequential catalytic cycles the 5W-BiVO_4_ catalyst retained 75% of its catalytic activity. The moderate loss of the catalytic activity is progressive and can be attributed to the loss of material mass at each centrifugation–recovery–reuse step, and aggregation phenomena of the particles as reported previously for other BiVO_4_ particles [26,28].

## 4. On the Mechanism of Photocatalytic Oxygen Evolution

Previous theoretical calculations suggest that surface oxygen-vacancies enhance the electron density near the bottom of the conduction band of BiVO_4_ [24]. Moreover, Vo and W-doping may play an important role in the interfacial charge transfer, and in this way W-doping is interlinked with the Vo [19,24] and the enhancement of the photocatalytic electron-hole separation [19,20,24]. To verify the role of the oxygen-vacancies, herein we oxidized, i.e., by mild calcination under O_2_, the best performing 5 W-BiVO_4_ and 5 Zr-BiVO_4_ particles.

**Figure 8:**
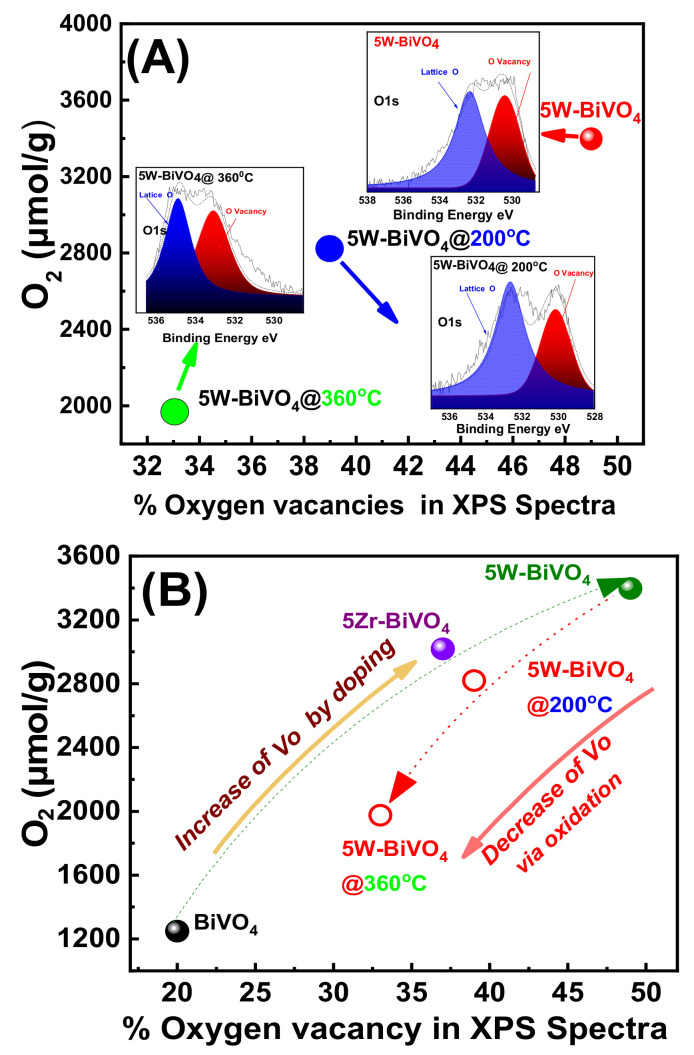
Photocatalytic O_2_-evolution evolution vs. the O-vacancies in the doped-BiVO_4_ catalysts. (**A**) Increase of O-vacancies is promoted by W- or Zr-doping in FSP-made BiVO_4_. Insert: O1s XPS spectra of the W-doped BiVO_4_. (**B**) Calcination under O_2_-rich atmosphere (oxidation) results in decrease of O-vacancies.

The data, see Figure 6C (open bars), show a sharp drop in O_2_-production efficiency as a function of the calcination temperature. The XRD data, see Figure S2 in Supporting Information, show an increase of particle size, i.e., it becomes 37 nm upon calcination at 360 °C for 3 h. At the same time, the Raman spectra, Figure S3 in Supporting Information, show a restoration of the peak position of the V-O(s) mode at 830 cm^−1^ indicating the elimination of the O-vacancies from the lattice of W-BiVO_4_. Moreover, there is a strong correlation of calcination with the decline of the O-vacancies population as detected by XPS, see Figure 8. An analogous phenomenon has been reported by other researchers also [21,24,25,31]. Here, our data allow a better comprehension of the necessity of W-doping and O-vacancies occurrence for enhancing O_2_-evolution. W-doping alone, i.e., without O-vacancies does not suffice for enhanced O_2_-evolution. Notice that in Figure 6C, after calcination at 360 °C the efficiency of the best-performing 5W-BiVO_4_ dropped below the non-doped BiVO_4_.

In agreement with previous reports, [31], the present data show that the primary factor for enhanced O_2_-evolution is the optimal O-vacancy engineering. Herein the 5W-BiVO_4_ and 5 Zr-BiVO_4_ materials, when oxidized by calcination at mild temperatures 200 °C and 360 °C for 3 h under air their O_2_-production declined from 3326 μmol/g to 2804 μmol/g and 1973 μmol/g. Analogous results observed for the 5 Zr-BiVO_4_ (see Table 2, and Figure 6).

## 5. Conclusions

The present research bears two key-results (i) FSP-technology allows engineering of oxygen vacancies content in BiVO_4_ via W-doping or Zr-doping, and (ii) oxygen-vacancies are the primary factor to determine the photocatalytic O_2_-production via water oxidation. FSP allows engineering of BiVO_4_ nanoparticles, with controlled heteroatom-dopant content, and oxygen vacancy content. As a proof of concept, W- and Zr-doped BiVO_4_ photocatalysts prepared with different dopant loading were engineered. The correlation between the catalytic efficiency and the oxygen-vacancies content provides strong evidence that the presence of oxygen vacancies in W-BiVO_4_, Zr-BiVO_4_ improves drastically the O_2_ production efficiency. Our present data on doped BiVO_4_ corroborate the mechanistic suggestions, indicating that the beneficial role of oxygen vacancy is a general phenomenon, which should be operating in various photocatalysts.

## Figures and Tables

**Table 1:** Properties of the flame spray pyrolysis (FSP)-made BiVO_4_ nanocatalysts *.

Material	Nominal Dopant Content in the FSP Precursor (%w:w)	Dopant Content Measured by XRF	XRD Size (nm ± 0.5)	SSA (m^2^g^−1^ ± 0.8)	Band-Gap Eg (eV)
BiVO_4_	-	-	22.1	37.7	2.36
W-Doped nanocatalysts					
0.5W-BiVO_4_	0.5	Not detectable	17.5	45.0	2.31
1W-BiVO_4_	1	0.6	19.8	46.8	2.25
2W-BiVO_4_	2	1.8	15.3	45.0	2.21
5W-BiVO_4_	5	4.4	17.1	47.2	2.14
10W-BiVO_4_	10	7.8	-	49.0	2.13
Zr-Doped nanocatalysts					
0.5Zr-BiVO_4_	0.5	Not detectable	17.8	47.1	2.27
1Zr-BiVO_4_	1	0.4	20.2	45.6	2.25
2Zr-BiVO_4_	2	1.7	19.8	45.7	2.20
5Zr-BiVO_4_	5	3.4	20.5	46.0	2.16
10Zr-BiVO_4_	10	8.3	-	47.2	2.15

* Data based on average of three batches.

**Table 2:** O_2-_evolution by the present FSP-made BiVO_4_-based photocatalysts.

Material	O_2_-Evolution (μmol/g) after 3 h Reaction	O_2_-Evolution Rate (μmol/g × h)
BiVO_4_	1249 (±20)	420 (±5)
W-Doped nanocatalysts		
0.5W-BiVO_4_	1842 (±20)	633 (±5)
1W-BiVO_4_	2002 (±20)	667 (±5)
2W-BiVO_4_	2827 (±20)	945 (±5)
5W-BiVO_4_	3326 (±20)	1020 (±5)
10W-BiVO_4_	2100 (±20)	778 (±5)
Zr-Doped nanocatalysts		
0.5Zr-BiVO_4_	600 (±20)	235 (±5)
1Zr-BiVO_4_	1039 (±20)	337 (±5)
2Zr-BiVO_4_	1512 (±20)	521 (±5)
5Zr-BiVO_4_	3018 (±20)	974 (±5)
10 Zr-BiVO_4_	907 (±20)	324 (±5)
Calcined nanocatalysts		
5W-BiVO_4_@200	2804 (±20)	1001 (±5)
5W-BiVO_4_@360	1973 (±20)	720 (±5)
5Zr-BiVO_4_@200	1988 (±20)	710 (±5)
5Zr-BiVO_4_@360	1299 (±20)	448 (±5)

**Table 3:** Comparison of the O_2_ evolution rate from BiVO_4_-based photocatalysts.

Photocatalyst	Light Source	Synthesis Method	XRD Size (nm)	Electron Acceptor	Activity (μmol/g*h)	Ref.
BiVO_4_	400 W medium-pressure halide lamp (Phillips HPA400, λ_max_ = 360 nm	Hydrothermal method	28.3	AgNO_3_	2622	[26]
BiVO_4_	300 W Xe lamp with a 420 nm cut off filter	Flame Synthesis of BiVO_4_ and Acid Modification	72.0	AgNO_3_	333	[27]
BiVO_4_	300 W Xe lamp with a 420 nm cut off filter	Solid-liquid state reaction in HNO_3_	91.0	AgNO_3_	800	[28]
Ce-BiVO_4_	300 W Xe lamp	Hydrothermal method	Not referred	AgNO_3_	775	[14]
A-FeOOH/BiVO_4_ (8 wt %)	300 W Xe lamp with a 420 nm cut off filter	Precipitation method	Not referred	NaIO_4_	1206	[29]
Zr-BiVO_4_	Perkin Elmer CERMAX LX-300BUV Xe lamp	Precipitation method	Not referred	AgNO_3_	700	[13]
W(0.9%w/w)-BiVO_4_	Simulated plasma (Lumix model LIFI STA-40)	Hydrothermal method	92.0	AgNO_3_	942	[30]
BiVO_4_	125W Hg λ_max_ = 440 nm	FSP	22.1	AuHCl_4_	420	This work
5W-BiVO_4_	125W Hg λ_max_ = 440 nm	FSP	17.1	AuHCl_4_	1074	This work
5Zr-BiVO_4_	125W Hg λ_max_ = 440 nm	FSP	20.5	AuHCl_4_	1020	This work

## Data Availability

Data is available upon the reasonable request from the corresponding author.

## Supplementary Materials

The following are available online at https://www.mdpi.com/2079-4991/11/2/501/s1, Figure S1: theoretical simulation of XRD patterns, Figure S2: XRD data for W doped BiVO_4._ Figure S3: Raman spectra of calcined W doped BiVO_4_.
