# Supplementary material for: Lattice Defects Engineering in W-, Zr-doped BiVO4 by Flame Spray Pyrolysis: Enhancing Photocatalytic O2 Evolution
Source: Nanomaterials (Basel). 2021 Feb 16;11(2):501. doi: 10.3390/nano11020501 (PMC7920441; doi:10.3390/nano11020501)
Supplement: Supplementary file 1 [file nanomaterials-11-00501-s001.pdf]

nanomaterials

Article

Lattice Defects Engineering in W-, Zr-doped $BiVO_4$ by Flame Spray Pyrolysis: Enhancing Photocatalytic O_2 Evolution

Panagiota Stathi, Maria Solakidou and Yiannis Deligiannakis *

Laboratory of Physics Chemistry of Materials & Environment, Department of Physics, University of Ioannina, Ioannina, 45110 , Greece; pstathi@cc.uoi.gr (P.S.); maria-sol@windowslive.com (M.S.)
* Correspondence: ideligia@uoi.gr

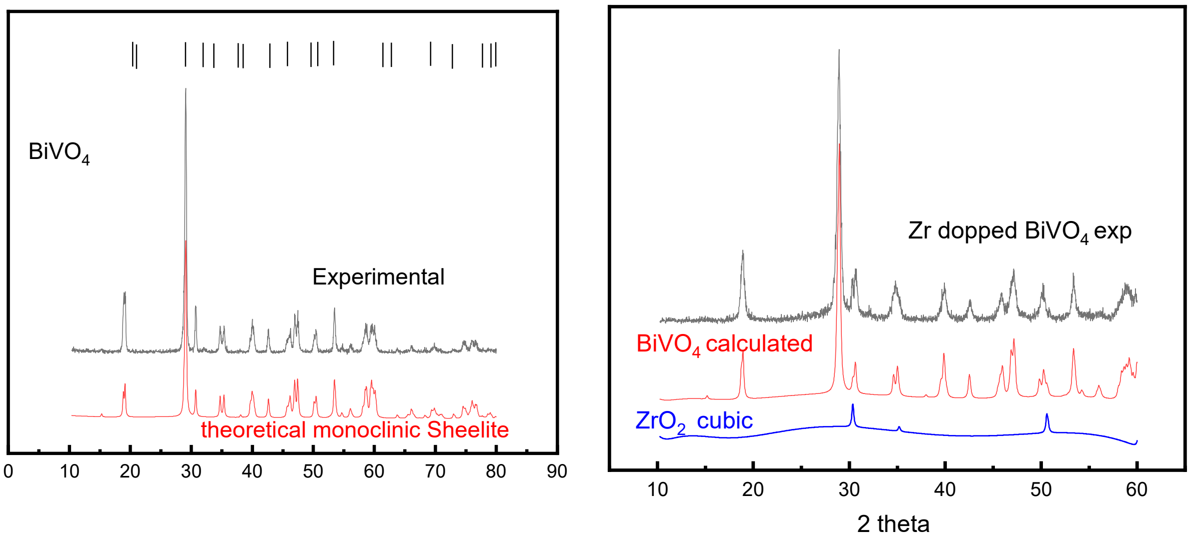

Figure S1. Theoretical analysis of XRD patterns of $BiVO_4$ and Zr-$BiVO_4$.

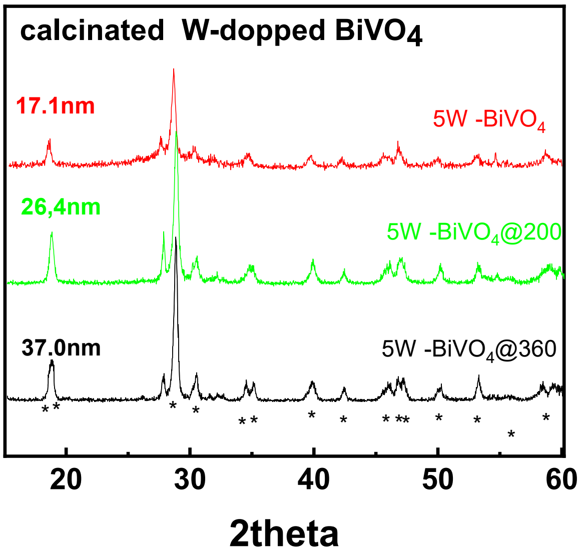

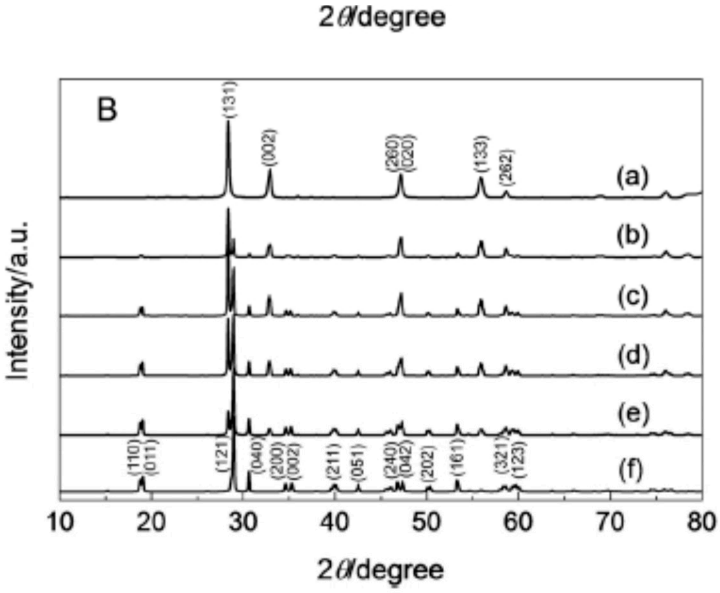

Figure S2. XRD pattern of calcined W-doped $BiVO_4$.

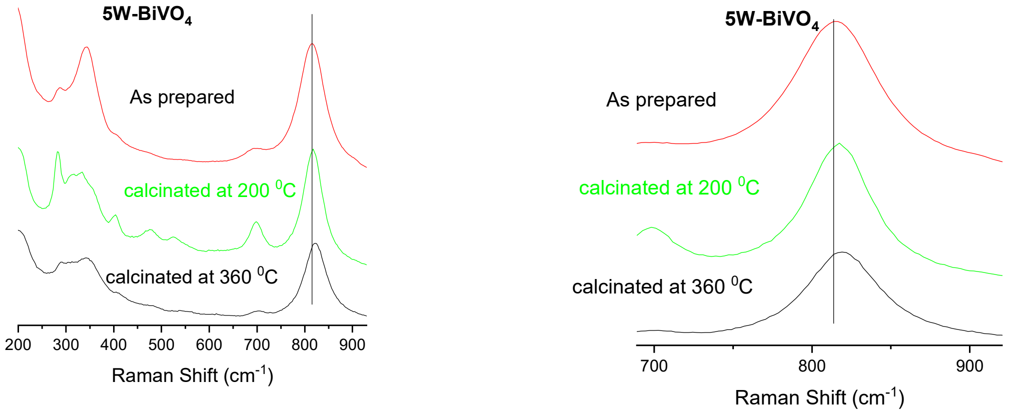

Figure S3. Raman spectra of calcined W-doped $BiVO_4$.
